# Fusogenicity of the Ghana Virus (*Henipavirus*: *Ghanaian bat henipavirus*) Fusion Protein is Controlled by the Cytoplasmic Domain of the Attachment Glycoprotein

**DOI:** 10.3390/v11090800

**Published:** 2019-08-29

**Authors:** Kathleen Voigt, Markus Hoffmann, Jan Felix Drexler, Marcel Alexander Müller, Christian Drosten, Georg Herrler, Nadine Krüger

**Affiliations:** 1 Institute of Virology, University of Veterinary Medicine Hannover, 30559 Hannover, Germany; 2 Research Center for Emerging Infections and Zoonoses, University of Veterinary Medicine Hannover, 30559 Hannover, Germany; 3 Infection Biology Unit, German Primate Center—Leibniz Institute for Primate Research, 37077 Göttingen, Germany; 4 Institute of Virology, Charité Universitätsmedizin Berlin, corporate member of Freie Universität Berlin, Humboldt-Universität zu Berlin and Berlin Institute of Health, 10117 Berlin, Germany

**Keywords:** Ghana virus, attachment glycoprotein, fusion, intracellular retention

## Abstract

The Ghana virus (GhV) is phylogenetically related to the zoonotic henipaviruses Nipah (NiV) and Hendra virus. Although GhV uses the highly conserved receptor ephrin-B2, the fusogenicity is restricted to cell lines of bat origin. Furthermore, the surface expression of the GhV attachment glycoprotein (G) is reduced compared to NiV and most of this protein is retained in the endoplasmic reticulum (ER). Here, we generated truncated as well as chimeric GhV G proteins and investigated the influence of the structural domains (cytoplasmic tail, transmembrane domain, ectodomain) of this protein on the intracellular transport and the fusogenicity following coexpression with the GhV fusion protein (F). We demonstrate that neither the cytoplasmic tail nor the transmembrane domain is responsible for the intracellular retention of GhV G. Furthermore, the cytoplasmic tail of GhV G modulates the fusogenicity of GhV F and therefore controls the species-restricted fusogenicity of the GhV surface glycoproteins.

## 1. Introduction

Viral RNA of the Ghana virus (GhV, formerly termed Kumasi virus, GenBank accession no.: HQ660129.1) was detected in a straw-colored fruit bat (*Eidolon helvum*) in 2009 [1]. GhV is phylogenetically related to Hendra (HeV) and Nipah (NiV) virus, two highly pathogenic members of the genus *Henipavirus* within the Paramyxoviridae family. HeV and NiV emerged from their reservoir host pteropid bats [2,3,4] causing severe and often fatal diseases in humans and domestic animals such as pigs and horses [5,6,7,8]. Due to their zoonotic potential, their high pathogenicity, and the lack of approved therapeutics and a vaccine licensed for use in humans, HeV and NiV are classified as biosafety level 4 pathogens. The zoonotic potential of GhV is unknown and so far, no cases of henipavirus infections of livestock or humans have been reported in Africa; but nevertheless, serological studies provided evidence that fruit bats, domestic animals, and humans in this region harbor neutralizing antibodies reactive against henipaviruses [1,9,10,11,12,13].

Henipaviruses express two surface glycoproteins: the fusion (F) and the attachment glycoprotein (G) that mediate the viral entry process. The G protein is a type II membrane protein and is responsible for binding to the cellular receptor ephrin-B2 [14,15,16,17,18,19]. The F protein, a type I membrane protein, mediates the fusion between viral and cellular membranes as well as the fusion between infected and neighboring cells to enable the release of the viral genome into the cytoplasm and to facilitate the spread of infection, respectively [20]. After synthesis, the F protein is transported to the cell surface as an inactive precursor that has to undergo a recycling step via clathrin-mediated endocytosis to be proteolytically cleaved by the cellular proteases cathepsin L or B within the endosomal compartment [21,22,23,24,25]. Furthermore, the F protein has to undergo conformational changes that are triggered by the interaction between F and G [26,27,28]. Upon binding to the cellular receptor, the G protein undergoes three conformational changes in the head and stalk regions that induce the refolding of F into its fusion-active conformation [29,30].

To gain information about the functionality of the two surface glycoproteins of GhV, previous studies focused on the directed expression of these two viral proteins in cell culture systems, as neither viral isolate nor recombinant GhV are available. In this way, it has been shown that the GhV G protein interacts with ephrin-B2, the cellular receptor of NiV and HeV, to mediate binding to target cells [31,32,33,34]. However, striking differences between NiV and GhV were observed when the fusogenicity of F was investigated: In the case of NiV, coexpression of F and G resulted in the formation of multinucleated giant cells, so-called syncytia, in different mammalian cell lines. In contrast, the GhV F and G proteins induced significantly smaller syncytia the presence of which was further restricted to bat cell lines [32,33,34,35]. In previous studies, it has been shown that the transport of GhV G to the cell surface is significantly reduced compared to NiV G and that the majority of the GhV G protein accumulates in the endoplasmic reticulum (ER), suggesting that the amount of surface-expressed GhV G is not efficient enough to trigger conformational changes in the F protein that are required to acquire the fusion-active form of F [35,36].

In this study we addressed the question: which domain of GhV G contributes to the accumulation in the ER and to the reduced fusogenicity of GhV F? Therefore, we generated truncated as well as chimeric GhV G proteins and analyzed their intracellular localization and the fusogenicity of GhV F following coexpression. We provide evidence that the truncation of the cytoplasmic domain (CD) results in a GhV G that enhances fusion activity of GhV F and abolished the species restriction, though the cellular localization did not differ from that of wildtype GhV G. 

## 2. Materials and Methods 

### 2.1. Cell Lines

Vero 76 (African green monkey, kindly provided by Andrea Maisner), HEK 293T (Human, DSMZ no. ACC-635), BHK-21 (Human, DSMZ no. ACC-61), EidNi/41 (Straw-colored fruit bat, *Eidolon helvum*) [37], and HypNi/1.1 (Hammer-headed fruit bat, *Hypsignathus monstrosus*) [38] cells were maintained in Dulbecco’s modified Eagle’s medium (DMEM; ThermoFisher Scientific, Waltham, MA, USA) supplemented with 5% or 10% fetal calf serum (FCS; PAN-Biotech, Aidenbach, Germany) and 1% penicillin/streptomycin (PAA Laboratories GmbH, Pasching, Austria). Cells were cultivated in 75 cm² tissue culture flasks (Greiner Bio-One, Frickenhausen, Germany) at 37 °C and 5% CO_2_.

### 2.2. Expression Plasmids

**Figure 1:**
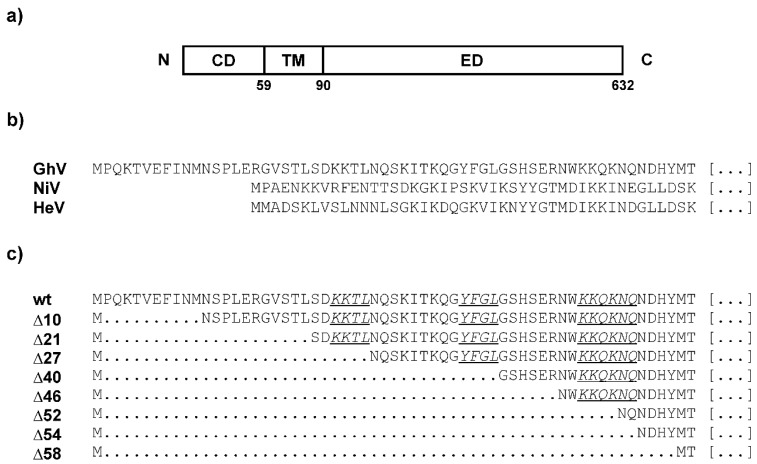
Organization of wildtype and truncated GhV G proteins. (**a**) Schematic illustration of the GhV G protein. CD: cytoplasmic domain, TM: transmembrane domain, ED: ectodomain. The TM was predicted using the online tool HMMTOP (www.enzim.hu/hmmtop/). (**b**) Amino acid sequence of the CD of NiV, HeV, and GhV G. (**c**) N-terminal amino acid sequences of GhV G wildtype (wt) and deletion mutants (∆10–∆58). Potential lysine- and tyrosine-based retention motifs are underlined and written in italics.

Expression plasmids coding for epitope-labelled full-length NiV and GhV F and G proteins have been described previously [33]. Here, we generated truncated versions of GhV G in which 10 to 58 amino acid (aa) residues of the N-terminus were deleted (GhV GΔ10, Δ21, Δ27, Δ40, Δ46, Δ52, Δ54, Δ58) (Figure 1). Furthermore, chimeric G proteins consisting of the transmembrane domain (TM) and ectodomain (ED) of GhV G and the cytoplasmic domain (CD) of NiV G (GhV G CD-NiV), HeV G (GhV G CD-HeV), Moijang henipavirus (MojV) G (GhV G CD-MojV), Cedar henipavirus (CedV) G (GhV G CD-CedV), or Measles virus (MeV) H protein (GhV G CD-MeV) were generated. In addition, NiV G TM and ED were fused to the CD of GhV G (NiV G CD-GhV). Further, we generated chimeric GhV and NiV G proteins in which the TM was replaced by the corresponding section of the other protein (GhV G TM-NiV, NiV G TM-GhV). Proteins consisting of GhV G CD and TM and the ED of NiV G (GhV G ED-NiV) and vice versa (NiV G ED-GhV) have been described previously [35]. To allow detection by immunostaining, all G proteins were fused to a FLAG-epitope at the C-terminal end, while NiV F and GhV F contained an HA-epitope at their corresponding C-terminus. All plasmids were verified by automated sequencing (Eurofins Genomics GmbH, Ebersberg, Germany). To determine the intracellular localization, cotransfection experiments with fluorescence-labeled cellular compartment markers for the endoplasmic reticulum (ER) were performed (EGFP-ER, provided by Frank van Kuppeveld).

### 2.3. Immunofluorescence Analysis

Cells were grown on coverslips and transfected using the ICAFectin^TM^ 441 transfection reagent (In-Cell-Art, Nantes, France) according to the manufacturer’s protocol. Cells were fixed with 3% paraformaldehyde (PFA) and either permeabilized with 0.2% Triton X-100 or untreated to detect intracellular or surface-expressed G proteins, respectively. F and G proteins were detected by incubation with antibodies directed against the HA- (dilution 1:500, rabbit, Sigma-Aldrich, St. Louis, MO, USA) or the FLAG-epitope (dilution 1:500, mouse, Sigma-Aldrich, St. Louis, MO, USA), respectively. Next, the incubation with anti-mouse IgG Alexa Fluor 568- (dilution 1:1000, Life Technologies, Carlsbad, CA, USA) and anti-rabbit IgG fluorescein isothiocyanate-conjugated (dilution 1:1000, Sigma-Aldrich, St. Louis, MO, USA) secondary antibodies was performed. Finally, the cells were incubated with 4′,6-diamidino-2-phenylindole (DAPI; Carl Roth GmbH + Co. KG, Karlsruhe, Germany) and mounted in ProLong Gold antifade reagent (Thermo Fisher Scientific, Waltham, MA, USA). Immunofluorescence analysis was performed using a Nikon Eclipse Ti microscope and NIS Elements AR software (Nikon Instruments, Tokyo, Japan).

To analyze the fusogenicity following co-expression of F and G proteins, the total number of syncytia per coverslip as well as the number of nuclei in each syncytium were counted. Based on these two parameters, the cumulative number of nuclei in syncytia per coverslip was calculated and used as an indicator for fusogenicity. Syncytia were defined as multinucleated cells that are positive for the expression of both viral proteins and harbor at least three nuclei.

### 2.4. SDS-PAGE and Western Blotting

HEK 293T cells were grown in 6-well plates and transfected for the expression of G proteins by calcium–phosphate precipitation. At 42 h p.t., cells were washed and resuspended in PBS, transferred to reaction tubes, and pelleted by centrifugation at 1000× *g* for 10 min at 4 °C. The cells were subsequently resuspended in NP40 lysis buffer with protease inhibitors and incubated on ice for 30 min. Afterwards, the cell lysates were centrifuged for 30 min at 10,000× *g* and 4 °C. The supernatant was transferred to a new reaction tube and mixed with 4× LDS sample buffer (Life Technologies) in a ratio of 1 to 4. Subsequently, dithiothreitol (DTT, 0.1 M final concentration) was added and the samples were heated for 10 min at 96 °C, before the lysates were loaded on an SDS gel (10% SDS) and further subjected to SDS-PAGE and Western blotting. G proteins were detected by incubation with an anti-FLAG antibody (dilution 1:500, mouse, Sigma-Aldrich, St. Louis, MO, USA) and anti-mouse horseradish peroxidase (HRP, dilution 1:1000, Dako GmbH, Jena, Germany). For the visualization of protein bands, membranes containing the immobilized proteins were incubated with Super Signal West Dura extended duration substrate (Thermo Fisher Scientific, Waltham, MA, USA), placed in a ChemiDoc imager (Bio-Rad, Hercules, CA, USA), and analyzed with the Image Lab Software (Bio-Rad, Hercules, CA, USA).

### 2.5. Co-Immunoprecipitation

HEK 293T cells were grown in 6-well plates and transfected for the expression of G proteins by calcium–phosphate precipitation. At 42 h p.t., cells were lysed as described above. Cell lysates were loaded onto 50 µL of protein-A sepharose beads (Sigma-Aldrich, St. Louis, MO, USA) mixed with recombinant mouse ephrin-B2-Fc (R&D Systems, Minneapolis, MN, USA) and incubated overnight at 4 °C on an overhead shaker. The samples were centrifuged for 3 min at 16,000 g and 4 °C. The supernatant was discarded, and the pellet was washed by the addition of 500 µL NP40 lysis buffer. This washing procedure was repeated three times. Afterwards, 50 µL of 1× LDS sample buffer (Life Technologies, Carlsbad, CA, USA) were added to the pellet and incubated for 10 min at 96 °C. Finally, the samples were centrifuged for 5 min at 13,000 rpm at room temperature, before the supernatants were loaded onto an SDS gel. SDS-PAGE, Western blotting, and protein detection by incubation with an anti-FLAG antibody and anti-mouse HRP were performed as mentioned before.

### 2.6. Flow Cytometry

HEK 293T cells were grown in 6-well plates and transfected for the expression of G proteins by calcium–phosphate precipitation. At 42 h p.t., cells were washed and resuspended in PBS, transferred to reaction tubes, and pelleted by centrifugation at 600× *g* for 10 min at 4 °C. The cells were subsequently resuspended in FACS-buffer (PBS without calcium and magnesium ions (PBSM)/1% bovine serum albumin (BSA)/3 mM EDTA) and centrifuged again before they were resuspended in FACS-buffer containing anti-FLAG antibodies (dilution 1:500, mouse, Sigma-Aldrich, St. Louis, MO, USA), followed by incubation for 1 h on an overhead shaker at 4 °C. Afterward, the cells were washed. For this, the cells were pelleted by centrifugation and resuspended in FACS-buffer. Then the cells were pelleted again, resuspended in FACS-buffer containing biotin-conjugated anti-mouse antibodies (dilution 1:500, Sigma-Aldrich, St. Louis, MO, USA), and rotated for 30 min at 4 °C. After an additional washing step, cells were resuspended in FACS-buffer containing phycoerythrin-conjugated streptavidin (dilution 1:1000, Bio-Rad, Hercules, CA, USA) and rotated for 30 min at 4 °C. Next, the cells were washed and subsequently fixed by incubation with 3% PFA for 20 min. Finally, the cells were washed again to remove residual PFA and resuspended in 300 µL FACS-buffer, before the mean fluorescence intensity was quantified by flow cytometry using an Attune NxT Flow Cytometer (Thermo Fisher Scientific, Waltham, MA, USA). The data analysis was performed by FlowJo® (FlowJo LLC, Ashland, OR, USA). 

## 3. Results

### 3.1. Truncation of the Cytoplasmic Domain of GhV G Enhances the Fusogenicity of GhV F and Abolishes Host Species-Restricted Cell-to-Cell Fusion

The GhV G protein consists of three major domains: the N-terminal cytoplasmic domain (CD), the transmembrane domain (TM), and the C-terminal ectodomain (ED). The latter can be subdivided into a stalk and a head domain (Figure 1a). Despite a similar structural organization, the CD of GhV G is longer compared to the CD of NiV and HeV (61 vs 45 aa residues, respectively, Figure 1b). In general, the sequence of this domain is not well conserved among the henipavirus species ranging from approximately 40 to 63% aa identity (Table 1). To determine whether the CD of GhV G—in particular the three potential retention motifs—may contribute to the intracellular retention of this protein and thereby lead to the limited fusogenicity of GhV F and G in comparison to coexpression of NiV F and G, truncated versions of GhV G were generated in which up to 58 aa residues of the CD were deleted in a stepwise fashion (Figure 1c).

**Figure 2:**
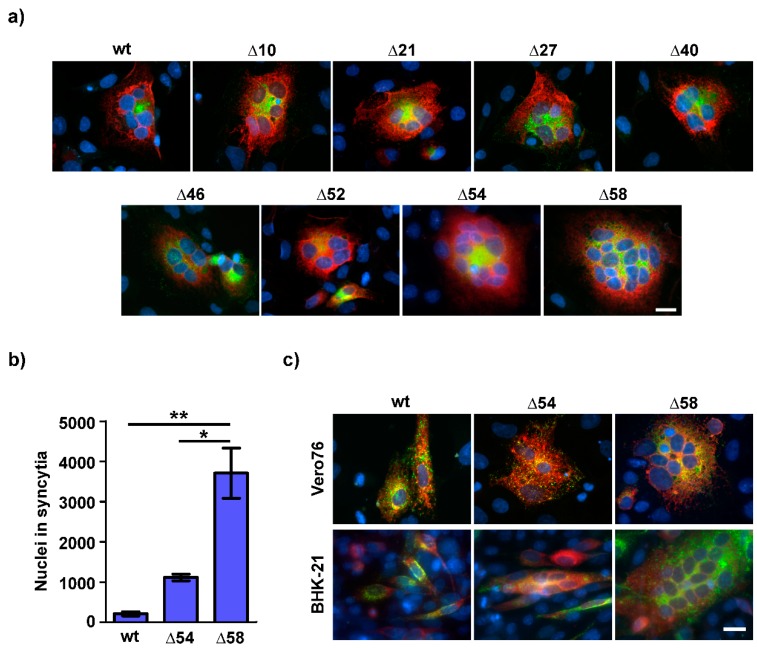
Truncation of the CD of GhV G results in an increased fusogenicity in bat and non-bat cells. (**a**) Coexpression of GhV F and wildtype or truncated G proteins in EidNi/41 cells. At 24 h p.t., cells were fixed, permeabilized, and incubated with antibodies for the detection of F and G proteins. Green: F, red: G, blue: nuclei. Scale bar indicates 20 µm. (**b**) The total number of nuclei in syncytia per coverslip was counted in GhV F and G (wildtype, ∆54 or ∆58) cotransfected EidNi/41 cells. The graph shows mean and SEM of three independent trials. Statistical significance was tested by paired, two-tailed Student’s t-test with 95% confidence intervals (*: *p* ≤ 0.05; **: *p* ≤ 0.01). A syncytium was defined as a multinucleated cell with positive staining for GhV F and G, containing at least three nuclei. (**c**) Coexpression of GhV F and wildtype or truncated G proteins in Vero76 and BHK-21 cells. Green: F, red: G, blue: nuclei. Scale bar indicates 20 µm.

Differences with respect to syncytium formation were obtained when the truncated G proteins were coexpressed with GhV F in bat (EidNi/41) and non-bat (Vero76 and BHK-21) cells. Consistent with previous publications, coexpression of wildtype GhV F and G mediates syncytium formation only in bat cell lines [32,33,34,35] and the truncated G proteins GhV G∆10, ∆21, ∆27, ∆40, ∆46, and ∆52 displayed the same phenotype as the full-length GhV G protein. In contrast to that, the deletion of the terminal 54 and 58 aa residues of the CD of GhV G resulted in an enhanced fusogenicity in bat cells as indicated by the increased number as well as the increased size of syncytia (Table 2, Figure 2a). This effect was most pronounced in the case of GhV G∆58 (Figure 2b). Most remarkably and in contrast to the parental G protein, GhV G∆54 and GhV G∆58 were able to induce cell-to-cell fusion also in the non-bat cell lines Vero76 and BHK-21 (Figure 2c).

### 3.2. Truncation of the Cytoplasmic Domain of GhV G Does not Affect Its Cellular Transport and Expression Pattern

**Figure 3:**
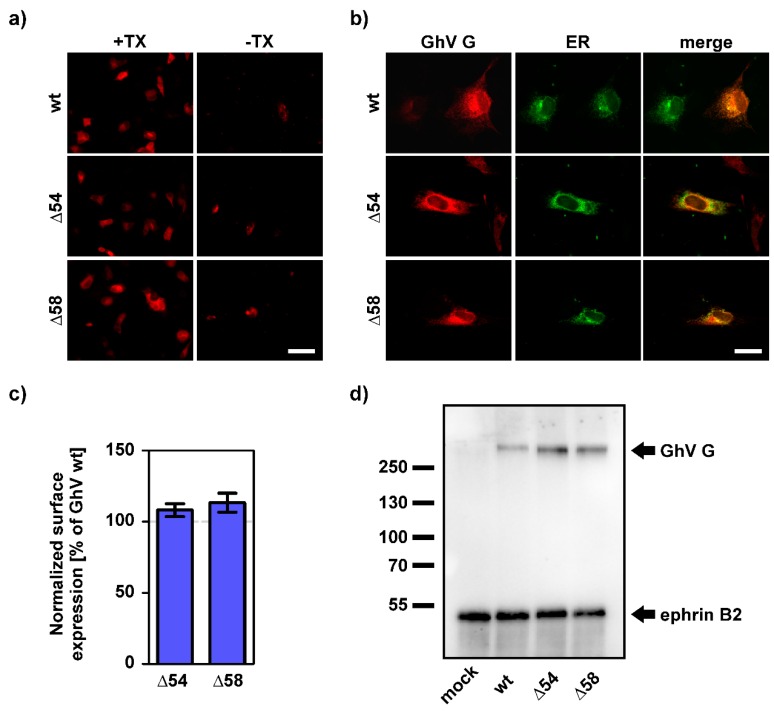
Truncation of the CD of GhV G does not affect the cellular expression pattern. (**a**) Fruit bat (EidNi/41) cells were transfected for the expression of wildtype or truncated GhV G proteins. At 24 h p.t., cells were either permeabilized (+TX) or non-permeabilized (-TX) to visualize total or only surface expressed proteins, respectively. Scale bar indicates 50 µm. (**b**) Fruit bat (HypNi/1.1) cells were cotransfected for the expression of GhV G proteins and a cellular compartment marker for the endoplasmic reticulum (ER). Red: G proteins, green: ER. Scale bar indicates 20 µm. (**c**) Surface expression of truncated GhV G proteins was quantified by flow cytometry and the mean fluorescence intensity obtained for GhV GΔ54 and Δ58 was normalized to that of wildtype GhV G, which was set as 100%. The graph shows mean and SEM of three independent trials. Statistical significance was tested by paired, two-tailed Student’s t-tests with 95% confidence intervals (no significant differences were obtained: *p* > 0.05). (**d**) Cell lysates of G expressing cells were loaded onto protein-A sepharose beads mixed with Fc-conjugated mouse ephrin-B2. After immunoprecipitation, SDS-PAGE and Western blotting were performed. Proteins were visualized by incubation with anti-Flag antibodies (mouse) and anti-mouse IgG horseradish peroxidase (HRP). Numbers on the left indicate molecular weight (kDa). Exposure time: 45 sec.

Similar to NiV and HeV G, the CD of GhV G contains two lysine-rich motifs at the aa positions 25–28 (KKTL) and 50–55 (KKQKNQ) and a tyrosine-based motif (37–41 YFGL), all of which have been implicated to be involved in the retention of diverse proteins within the ER [39,40] (Figure 1b). By truncation of the CD of GhV G we obtained modified versions that lacked either the first lysine-rich motif (GhV GΔ27), the first lysine-rich motif and the tyrosine-based motif (GhV G Δ40, Δ46), or all three motifs combined (GhV GΔ52, Δ54, Δ58) (Figure 1c). To investigate whether these motifs contribute to ER-retention, the surface expression levels and the intracellular expression pattern were analyzed. All truncated G proteins showed a total expression level comparable to that of full-length GhV G as indicated by similar amounts of fluorescence-positive cells (analyzed by immunofluorescence analysis of permeabilized cells) and no increased surface expression was observed for any of the G proteins as exemplarily shown for GhV GΔ54 and Δ58 (Figure 3a). Furthermore, the truncation of the CD did not affect the intracellular localization and all truncated versions of GhV G showed a clear colocalization with the ER marker, indicating that they are still intracellularly retained (Figure 3b). When comparing the surface expression level of full-length GhV G to that of GhV GΔ54 and Δ58 by flow cytometry, no significant differences were obtained (Figure 3c) although the latter proteins increased the fusogenicity of GhV F (Figure 2). In addition, to investigate whether the truncation affects the conformation of GhV G, the ability of truncated G proteins to interact with the receptor ephrin-B2 as well as their ability to dimerize has been analyzed. Both truncated G proteins were able to form high-order multimers and to bind to ephrin-B2 (Figure 3d).

### 3.3. Switching the CD of GhV G with that of Related or Unrelated Paramyxovirus G Proteins Does Not Affect Fusogenicity of GhV F 

**Figure 4:**
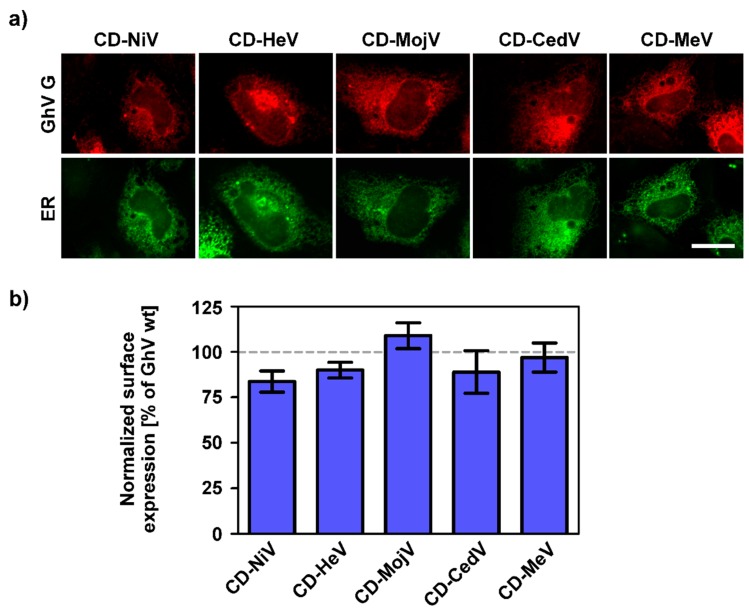
Replacement of the CD of GhV G does not increase the surface expression level. (**a**) Fruit bat (HypNi/1.1) cells were cotransfected for the expression of GhV G proteins harboring the CD of NiV, HeV, MojV, CedV, or MeV and a cellular compartment marker for the endoplasmic reticulum (ER). Red: G proteins, green: ER. Scale bar indicates 20 µm. (**b**) Surface expression of chimeric GhV G proteins was quantified by flow cytometry. The mean fluorescence intensity obtained for the chimeric GhV G proteins was further normalized to wildtype GhV G, which was set as 100%. The graph shows mean and SEM of three independent trials. Statistical significance was tested by paired, two-tailed Student’s t-tests with 95% confidence intervals (no significant differences were obtained: *p* > 0.05).

**Figure 5:**
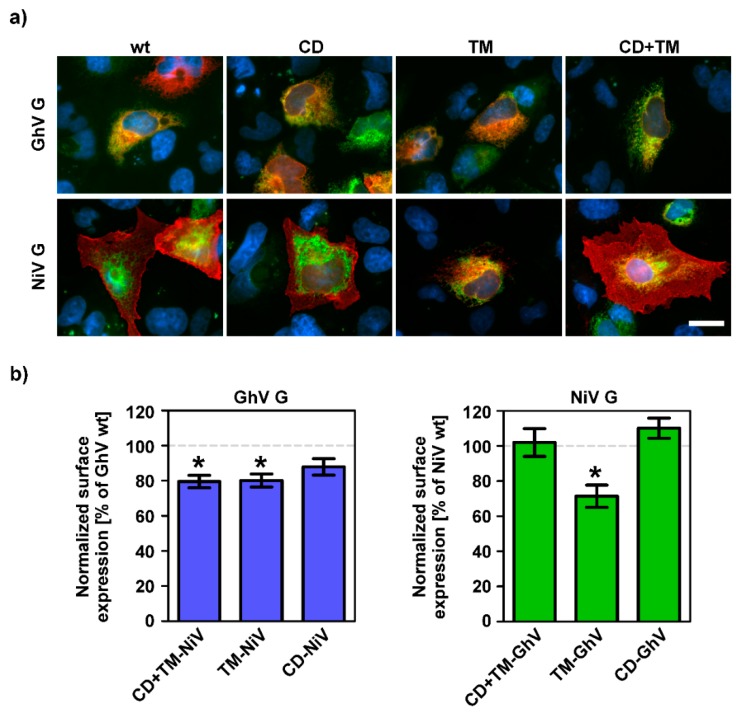
Replacement of GhV CD and TM does not increase the surface expression level of chimeric G proteins. (**a**) Fruit bat (HypNi/1.1) cells were transfected for the coexpression of wildtype or chimeric G proteins and a cellular compartment marker for the endoplasmic reticulum (ER). Red: G proteins, green: ER, blue: nuclei. Scale bar indicates 20 µm. (**b**) Surface expression of chimeric G proteins was quantified by flow cytometry. The mean fluorescence intensity obtained for chimeric G proteins was further normalized to that of the corresponding wildtype G proteins, which were set as 100%. The graph shows mean and SEM of three independent trials. Statistical significance of differences in surface expression of mutant versus wildtype G proteins was tested by paired, two-tailed Student’s t-tests with 95% confidence intervals (*: *p* ≤ 0.05).

After having shown that the truncated proteins GhV GΔ54 and Δ58 led to an increased fusogenicity of GhV F and abolished species restricted syncytium formation, we analyzed whether the replacement of the CD of GhV G by the corresponding domain of NiV G (GhV G CD-NiV) and vice versa (NiV G CD-GhV) has any effect on the fusogenicity of the respective F proteins. In addition, chimeric GhV G proteins harboring the CD of the G proteins of the related henipaviruses HeV, CedV, and MojV G, or the H protein of the unrelated paramyxovirus measles virus (MeV, genus *Rubulavirus*) were generated. All chimeric GhV G proteins showed an intracellular localization similar to that of the parental G protein (Figure 4a). Furthermore, the replacement of the CD did not increase the surface expression level of GhV G (Figure 4b). However, none of these G proteins was capable of inducing the fusion activity of coexpressed GhV F in bat or non-bat cell lines. In contrast, chimeric NiV G harboring the CD of GhV G showed an expression pattern comparable to that of parental NiV G (Figure 5a). Furthermore, coexpression of NiV G CD-GhV and NiV F resulted in the formation of large syncytia as observed after coexpression of parental NiV G and F (Table 3). The heterologous expression of chimeric GhV G and NiV F or chimeric NiV G and GhV F did not mediate syncytium formation.

### 3.4. The Exchange of the GhV G TM and CD Neither Supports Fusogenic Activity of GhV F Nor Controls GhV G Surface Expression

After replacing the CD of GhV and NiV G, we focused on the remaining domains and generated chimeric G proteins in which only the TM (GhV G TM-NiV, NiV G TM-GhV) or the TM in conjunction with the CD (GhV G CD + TM-NiV, NiV G CD + TM-GhV) were replaced. All chimeric G proteins were analyzed for their localization and their fusogenicity following coexpression with homo- or heterologous F proteins, given that the exchanged domains may be necessary for the specific interaction between the corresponding F and G proteins.

None of the chimeric G proteins was able to mediate cell-to-cell fusion regardless of the origin of the F protein or the cell line used (Table 3). All chimeric GhV G proteins showed the same expression pattern as parental GhV G and were predominantly localized in the ER (Figure 5a, upper lane). Further, the surface expression level of GhV G was slightly reduced by the replacement of any of the domains (Figure 5b). In contrast, the replacement of the TM of NiV G by the corresponding domain of GhV G led to a partial retention of the chimeric G protein within the ER (Figure 5a, lower lane) and significantly decreased the cell surface expression level (Figure 5b), which might have resulted from suboptimal compatibility of the three protein domains (CD, TM, ED) in this particular organization. Consistent with previous findings, no differences were detected when comparing the parental NiV G and NiV G CD + TM-GhV [35].

## 4. Discussion

A hallmark of the henipaviruses NiV and HeV is their strong fusion activity, which requires the interaction between the viral surface proteins F and G. However, whereas the glycoproteins of NiV and HeV induce cell-to-cell fusion in a broad variety of cells from different host species, syncytium formation upon expression of GhV F and G proteins is restricted to certain bat cells [32,33,34,35]. This difference was believed—at least in part—to be due to an inefficient surface transport of the G protein of GhV, as the restricted surface expression has been shown to be less pronounced in bat cells compared to non-bat cells [35]. Here we analyzed the importance of the functional domains of the G protein of GhV for GhV G surface transport and for triggering the fusogenic activity of GhV F.

Intracellular retention is also a characteristic feature of other viral glycoproteins and is often associated with intracellular budding of the respective enveloped viruses, such as coronaviruses, e.g., transmissible gastroenteritis virus, or pestiviruses, e.g., bovine viral diarrhea virus (BVDV) [41,42]. Further, viruses from different families express glycoproteins that are retained at intracellular structures, e.g., the envelope proteins E1 and E2 of rubella virus [43,44], the E2 and E^rns^ proteins of BVDV [45,46], the E1 protein of hepatitis C virus [47], the Gn and Gc proteins of Bunyamwera virus [48], or the spike glycoprotein (S) of certain coronaviruses [49,50,51,52]. For the latter, lysine- and/or tyrosine-based motifs have been identified to cause accumulation of the S protein within intracellular compartments of the secretory pathway [50,53]. The cytoplasmic tail of the GhV G protein contains two lysine-based motifs (25-KKTL-28 and 50-KKQKNQ-55) as well as a tyrosine-based motif (38-YFGL-41), but none of them appears to serve as a retention signal. This is evident from the comparison of the deletion mutants that lacked one, two, or all three motifs; none of them showed an increased surface expression. 

Retention in the ER may also be mediated by the TM domain of viral glycoproteins as shown for the E2 protein of BVDV [45,54]. Here an arginine residue within the hydrophobic amino acid sequence of the transmembrane anchor is crucial for the intracellular localization of this surface glycoprotein. However, the TM domain of the GhV G protein lacks a comparable amino acid sequence. When chimeric G proteins were analyzed, GhV G containing the TM of NiV-G did not show enhanced surface expression, while the TM of GhV in the context of NiV G led to an intracellular accumulation of the resulting G protein. Therefore, the information for the intracellular retention of GhV G resides mainly or exclusively within the ED. The presence of putative retention signals within the CD portion of G may raise the question whether these motifs may have a synergistic effect and support the retention signal in the ED. However, the results obtained with the chimeric proteins argue against this possibility, because they did not provide any evidence that CD of GhV-G contains an ER retention signal. Although the ultimate reason for intracellular retention of GhV G could not be uncovered, we could exclude a pivotal role of CD and TM for this retention. Moreover, our data suggest that the ED—more precisely, aa motifs/residues within the ED—might be the cause for GhV G accumulation within the ER. Once attempts to isolate GhV or to generate it by DNA technology have been successful, it will be interesting to find out whether the virus matures at the cell surface or whether the budding process takes place at an intracellular compartment. 

Other remarkable features of GhV surface glycoproteins that are distinct from NiV F and G are the dramatically reduced fusogenicity and the host restriction of cell-to-cell fusion upon coexpression of GhV F and G [32,33,34]. The less efficient fusion activity of the surface proteins of GhV—compared to that of the corresponding proteins of NiV—may be explained by differences in both the F [34] and the G protein [35]. As far as the G protein is concerned, a major difference between GhV and NiV is the aforementioned inefficient surface expression of GhV G. However, surface expression is not the only determinant of the G protein for interacting with the F-protein and inducing membrane fusion. Both the CD and the TM domain may affect the fusion activity, even in the absence of increased transport of the G protein to the cell surface. The importance of CD for the fusion activity is evident from our analysis of the deletion mutants GΔ54 and Δ58. Although the lack of the major part of the cytoplasmic tail did not result in a detectable increase of the surface expression or binding efficiency to ephrin B2, it enhanced the fusion activity in bat cells and more importantly, abolished the species restriction to bat cells. Thus, the CD portion has some restricting effect. It might affect the conformation of G that is required for the interaction with the F protein or it impedes conformational changes within the G protein that are necessary to render the F protein fusion-active [29,30]. This restriction appears to be a peculiar feature of GhV G because a chimeric protein containing ED and TM of NiV G and CD of GhV G was highly efficient in inducing the fusion activity of NiV F.

The results obtained with chimeric proteins further suggest that not only CD but also the TM domain affects the role of the G protein as a modulator of the fusion activity of henipavirus F proteins. While CD of GhV G did not affect the fusion activity of NiV glycoproteins, replacement of TM by the corresponding domain of GhV completely abolished the fusion activity of NiV F + G. As discussed for CD, the TM domain may affect the conformation of the G protein and thus the interaction with the F protein. An alternative explanation is that there is a direct interaction between the TM domains of the NiV F and G proteins and that this interaction does not tolerate the replacement of the NiV G-TM domain by GhV-G-TM.

In sum, we demonstrate that neither the CD nor TD cause intracellular retention of GhV G, which suggests that the structural cause for intracellular retention resides within the ED. Moreover, we identified the CD of GhV G as a modulator of GhV F fusogenicity and, most importantly, as the factor controlling species restriction of F-mediated membrane fusion. Future efforts will be undertaken to unravel which aa motif/residues are responsible for intracellular GhV G retention and whether the CD of GhV G (and other henipaviruses G proteins) is a key factor controlling the zoonotic potential. 

## Figures and Tables

**Table 1:** Amino acid identity of the cytoplasmic domain (CD) of henipavirus G proteins.

	GhV	NiV	HeV	CedV	MojV
**GhV**	-	47.6	42.6	55.6	40.4
**NiV**	47.6	-	63.2	49.8	41.8
**HeV**	42.6	63.2	-	42.6	43.6
**CedV**	55.6	49.8	42.6	-	41.8
**MojV**	40.4	41.8	43.6	41.8	-

GhV: Ghana virus, NiV: Nipah virus, HeV: Hendra virus, CedV: Cedar henipavirus, MojV: Moijang henipavirus. The aa identity was analyzed using the PRofile ALIgNEment (PRALINE) software (http://www.ibi.vu.nl/programs/pralinewww/).

**Table 2:** Syncytium formation following coexpression of GhV F and truncated G proteins.

Glycoprotein	Bat Cells	Non-Bat Cells
G wt	+	-
GΔ10	+	-
GΔ21	+	-
GΔ27	+	-
GΔ40	+	-
GΔ46	+	-
GΔ52	+	-
GΔ54	++	+
GΔ58	+++	++

Size of syncytia is indicated as the maximal number of nuclei per syncytium from three coverslips. (-) = no syncytia detected; (+) = ≤5 nuclei per syncytium; (++) = 5–10 nuclei per syncytium; and (+++) = ≥10 nuclei per syncytium.

**Table 3:** Syncytium formation following coexpression of GhV or NiV F and chimeric G proteins.

Glycoprotein	GhV F	NiV F
GhV G wt	+ ^1^	++
GhV G CD-NiV	-	-
GhV G TM-NiV	-	-
GhV G CD + TM-NiV	-	-
NiV G wt	-	+++
NiV G CD-GhV	-	+++
NiV G TM-GhV	-	-
NiV G CD + TM-GhV	-	-

Size of syncytia is indicated as the maximal number of nuclei per syncytium from three coverslips. (-) = no syncytia detected; (+) = ≤5 nuclei per syncytium; (++) = 5–10 nuclei per syncytium; and (+++) = ≥10 nuclei per syncytium. ^1^ Fruit bat cells only.
